# *Staphylococcus* *aureus* in Bovine Mastitis: Pathogenesis, Antimicrobial Resistance, and Emerging Control Strategies

**DOI:** 10.3390/microorganisms14051125

**Published:** 2026-05-15

**Authors:** Cosmina Maria Bouari, George Cosmin Nadăş, Smaranda Crăciun, Nicodim Iosif Fiț

**Affiliations:** Department of Microbiology, Immunology and Epidemiology, Faculty of Veterinary Medicine, University of Agricultural Sciences and Veterinary Medicine, 400372 Cluj-Napoca, Romania; cosmina.bouari@usamvcluj.ro (C.M.B.); smaranda.craciun@usamvcluj.ro (S.C.); nfit@usamvcluj.ro (N.I.F.)

**Keywords:** *Staphylococcus aureus*, bovine mastitis, antimicrobial resistance, biofilm, alternative therapies, intramammary infection, virulence factors, One Health

## Abstract

Bovine mastitis is a major infectious disease in dairy cattle, causing significant economic losses and compromising animal health and milk quality worldwide. Among its etiological agents, *Staphylococcus aureus* is a key contagious pathogen due to its ability to establish persistent intramammary infections and evade host immune responses and antimicrobial therapy. This review summarizes current knowledge on the epidemiology, pathogenesis, clinical presentation, diagnosis, and control of *S. aureus* in bovine mastitis. Particular emphasis is placed on virulence mechanisms, including adhesion, intracellular persistence, biofilm formation, and immune evasion, which contribute to chronic and recurrent infections. The increasing prevalence of antimicrobial resistance, including methicillin-resistant and multidrug-resistant strains, is highlighted as a major challenge limiting treatment efficacy and posing risks within a One Health context. The review also discusses emerging alternative therapies and innovative control strategies, such as anti-biofilm approaches, immunomodulation, and improved diagnostics, aimed at reducing antimicrobial use. Advances in molecular and point-of-care diagnostic tools are considered for their role in early detection and targeted interventions. Overall, effective control of *S. aureus* mastitis requires integrated strategies combining prudent antimicrobial use, alternative therapies, improved hygiene, and a multidisciplinary One Health approach.

## 1. Introduction

Mastitis remains one of the most pervasive and costly diseases in dairy cattle, with significant impacts on milk production, quality, and animal health worldwide. It is estimated that clinical and subclinical forms account for tens of billions in global losses each year, driven primarily by reduced milk yield, impaired milk quality, and increased management costs [1]. Because subclinical mastitis often goes undetected, its cumulative economic impact can exceed that of overt clinical cases in many herds [2]. Prevalence rates vary widely depending on region and management practices, with subclinical mastitis often exceeding 40–60% in intensively managed herds, highlighting its substantial but frequently underestimated impact [3,4,5]. Given this substantial toll, understanding pathogen-specific dynamics (such as those of *S. aureus*) is essential to refine control strategies and improve udder health.

Among the various bacterial agents implicated in bovine mastitis, *S. aureus* stands out as one of the most important contagious pathogens owing to its efficient cow-to-cow transmission and ability to establish persistent intramammary infections (IMI). In many dairy systems, *S. aureus* is transmitted primarily during milking via contaminated hands, teat liners, or milking equipment, making it harder to eliminate compared to many environmental pathogens [6]. Because *S. aureus* colonizes teat skin and ductal epithelium, and can exist in subclinical form for long periods, it often evades detection and spreads silently within herds [6,7].

Furthermore, genomic and epidemiological evidence confirms that *S. aureus* clones adapted to bovine hosts frequently dominate mastitis cases globally, with clonal complexes (e.g., CC97, CC8) showing widespread dissemination and strong contagious potential [8]. The ability of *S. aureus* to persist, even under milking hygiene and control programs, highlights its role as a high-priority pathogen in dairy mastitis research and control efforts [7].

Focusing on *S. aureus* in bovine mastitis is critical because this pathogen is notorious for causing persistent, chronic intramammary infections that are resistant to conventional therapies [7,8]. Its low bacteriological cure rates, even after standard antimicrobial treatment, stem from its ability to persist in mammary tissue, evade immune clearance, and periodically resurge [9]. Many mastitis outbreaks attributed to *S. aureus* linger across lactations, compelling farmers toward repeated treatments or culling of affected animals. Moreover, *S. aureus* often acquires multidrug resistance (MDR) or even extensively drug-resistant (XDR) profiles, mediated by mobile genetic elements, β-lactamase production, and *mecA*/SCCmec (Staphylococcal Cassette Chromosome mec) systems, further undermining therapeutic success [10,11].

Another compelling reason to spotlight *S. aureus* is its strong biofilm-forming capability, which shields bacterial communities inside extracellular polymeric matrices, reducing antibiotic penetration and protecting them against host immune effectors [12]. In many isolates from bovine mastitis, biofilm phenotypes correlate with virulence and resistance determinants, reinforcing the recalcitrant nature of infections [13]. Importantly, *S. aureus* exhibits a zoonotic and public health dimension: livestock-associated MRSA strains have been isolated in dairy workers, and contamination of raw milk or dairy products poses risks of cross-species transmission [14]. The confluence of chronicity, resistance, biofilm resilience, and zoonotic potential makes *S. aureus* a prime candidate for an in-depth, focused review in the context of bovine mastitis. Taken together, these factors indicate that the persistence and impact of *S. aureus* mastitis cannot be attributed to a single mechanism but rather to a complex interplay of microbial adaptation, host response, and management practices.

Unlike previous reviews that often address individual aspects of *S. aureus* mastitis—such as virulence mechanisms, antimicrobial resistance, diagnostics, or alternative therapies separately, the present review integrates these areas into a unified framework focused on persistence, therapeutic failure, and practical control challenges. By emphasizing recent findings, comparative trends, and translational gaps, this review provides a more critical synthesis of current knowledge and highlights priorities for future research and field implementation. To further clarify the scope and distinct contribution of this review in the context of existing literature, a comparative overview of recent reviews is presented in Table 1.

This review was conducted through a structured literature search focusing on *Staphylococcus aureus* and bovine mastitis. Peer-reviewed publications from January 2015 to February 2026 were retrieved from PubMed, Scopus, Web of Science, and Google Scholar using combinations of the following keywords: *Staphylococcus aureus*, bovine mastitis, biofilm, antimicrobial resistance, epidemiology, and One Health. Additional relevant reports from FAO, OIE, and WHO were considered to contextualize global trends. Earlier foundational studies published before 2015 were also incorporated where necessary to support key concepts and historical perspectives. Only English-language articles were included.

Titles and abstracts were screened for relevance, and full texts of key studies were reviewed to extract information on epidemiology, pathogenesis, resistance mechanisms, and control strategies. Emphasis was placed on recent findings (2018–2025), though seminal works predating this range were included where conceptually essential. Data were synthesized qualitatively to highlight thematic patterns, knowledge gaps, and emerging trends. References were managed using Zotero. Although elements of a structured literature search were applied, this manuscript is intended as a narrative review rather than a formal systematic or scoping review. Therefore, a PRISMA flow diagram and a detailed study selection process were not included.

This review has several limitations. The analysis was restricted to English-language publications, potentially introducing language bias. In addition, the defined time window may have led to the exclusion of relevant earlier studies, although key foundational work was included where necessary. The included studies are heterogeneous in terms of design, population, and diagnostic approaches, which may affect comparability. Furthermore, for many emerging therapeutic strategies, the available evidence remains limited and largely based on in vitro or early-stage experimental studies, which may constrain their immediate applicability in field conditions.

## 2. Epidemiology and Global Burden

Epidemiological studies indicate that the prevalence of *S. aureus* in bovine mastitis varies markedly between developed and developing regions, reflecting differences in herd management, diagnosis, and control practices. In some developing countries, reported prevalence figures are strikingly high: for example, in Ethiopia the apparent prevalence of *S. aureus* in mastitic cows ranged from 10% up to 66.6% across studies, depending on region and sampling methods [17]. In a recent study from Egypt and surroundings, *S. aureus* was isolated in ~44% of bacteriologically examined milk samples from clinical and subclinical mastitis cases [18]. Meanwhile, in developing Asia, *S. aureus* was detected in 42.5% of mastitis-positive samples in a survey with a higher incidence in subclinical than clinical cases (45.8% vs. 37.5%) [15].

By contrast, in many developed-country herds the prevalence of *S. aureus* intramammary infection is often lower, albeit with significant within-herd variability. For example, in dairy farms with low bulk-tank somatic cell count (SCC), cow-level prevalence of *S. aureus* is typically estimated at 1–10%, yet in herds with poorer udder hygiene it may reach 50–75% [6]. In Europe, a study across 10 European countries observed notable variation in the prevalence of intramammary *S. aureus* infections linked to specific genotypes such as adlb-positive strains [8]. A global meta-analysis of mastitis pathogens estimated that *S. aureus* accounts for approximately 25–28% of mastitis isolates overall (from data spanning multiple continents between 1979 and 2019) [19]. These disparities between regions highlight how management practices, surveillance intensity, and diagnostic methods influence observed prevalence and increase the importance of context when comparing *S. aureus* mastitis burdens across countries. These prevalence estimates should therefore be interpreted with caution, as they are derived from studies employing different sampling strategies, definitions of mastitis, and diagnostic methods, limiting direct comparability across regions and production systems. Moreover, such heterogeneity may lead to under- or overestimation of the true disease burden, highlighting the need for more standardized epidemiological approaches. To facilitate comparison of epidemiological data across regions, key findings from representative studies are summarized in Table 2.

The marked regional variation in *S. aureus* mastitis prevalence is driven by a complex interplay of animal-level, herd-level, and management factors, many of which are exacerbated under resource-limited conditions. At the cow level, older age, higher parity, and a history of previous mastitis episodes consistently emerge as risk factors [3,4,16]. For example, *S. aureus* infection rates increase in multiparous cows compared to primiparous ones, likely due to cumulative tissue damage, relaxed teat sphincters, and residual infections persisting across lactations [20]. Cows in later lactation stages also tend to harbor more subclinical *S. aureus* infections, reflecting the chronic and latent nature of many infections [3].

Beyond animal intrinsic factors, management and hygiene practices play a pivotal role, especially where infrastructure or training is limited. Key drivers include inadequate teat sanitation (poor pre- and post-milking teat washing or dipping), lack of hand hygiene between cows, and contaminated milking equipment [3,20]. Herd size and stocking density contribute by facilitating pathogen transmission: larger herds or overcrowded housing increase contact rates and stress, which can weaken immune defenses. Environmental factors such as poor housing ventilation, wet bedding, and manure accumulation also promote bacterial survival on surfaces and teats, enhancing infection risk. Finally, limited access to veterinary care or diagnostic services in developing regions often results in delayed detection and treatment, enabling *S. aureus* to persist and disseminate unchecked [2,3,4].

Transmission of *S. aureus* within dairy herds follows a primarily contagious pattern, with infection spreading from cow to cow, especially during milking [6]. The teat canal serves as the main portal of entry, and contaminated milking equipment, liners, or milkers’ hands are key vehicles for bacterial transfer [6]. Unlike environmental pathogens such as *E. coli* or *Klebsiella* spp., *S. aureus* typically resides in chronically infected udders, serving as a persistent source for new infections within the herd. Consequently, control strategies have long targeted reducing transmission during milking through strict hygiene, segregation of infected cows, and disinfection protocols [21,22].

Recent molecular epidemiological studies have revealed that *S. aureus* isolates from bovine mastitis often cluster within specific clonal complexes (CCs) that show adaptation to bovine hosts, notably CC97, CC705, CC151, and CC398 [7,23]. These lineages display genetic stability and are maintained in herds for years, consistent with chronic endemic transmission cycles. Notably, *S. aureus* CC398, initially described as a livestock-associated methicillin-resistant lineage (LA-MRSA), has increasingly been detected in dairy herds and humans working in close contact with cattle, suggesting bidirectional zoonotic exchange [24].

Furthermore, asymptomatic carriers, particularly heifers, dry cows, and replacement animals, may act as hidden reservoirs that sustain transmission across lactations [20,23]. Recent evidence indicates that contaminated bedding and milking cloths can also harbor viable *S. aureus* for several days, potentially facilitating mechanical spread in poorly sanitized facilities [25,26]. Together, these findings emphasize that *S. aureus* epidemiology is shaped by both microbial adaptation and herd-level management, reinforcing the need for biosecurity and early detection to break transmission chains.

## 3. Pathogenesis of *S. aureus* Mastitis

**Figure 1:**
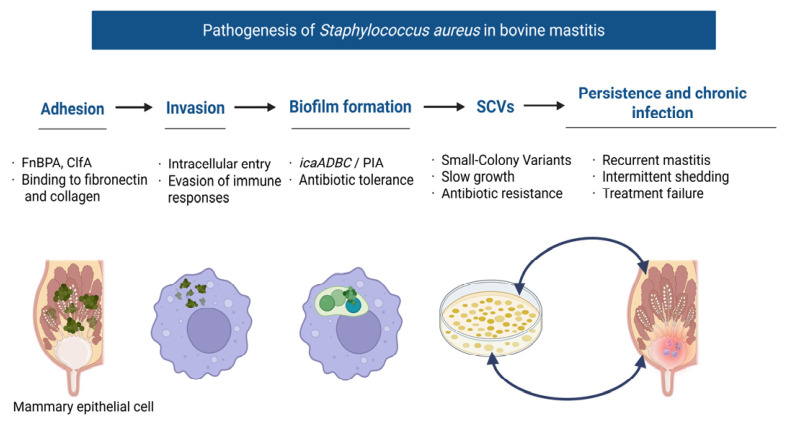
Pathogenesis of *Staphylococcus aureus* in bovine mastitis. The infection process involves sequential stages including adhesion to mammary epithelial cells via microbial surface components recognizing adhesive matrix molecules (MSCRAMMs), invasion and intracellular survival, biofilm formation mediated by the *icaADBC* operon and polysaccharide intercellular adhesin (PIA), and the emergence of small-colony variants (SCVs). These mechanisms contribute to immune evasion, antimicrobial tolerance, and the establishment of persistent and chronic intramammary infections. Abbreviations: FnBPs, fibronectin-binding proteins; ClfA, clumping factor A; icaABCD, intercellular adhesion gene cluster; PIA, polysaccharide intercellular adhesin; SCVs, small colony variants. Created in BioRender. Nadas, N. (2026) https://BioRender.com/dzl968q (accessed on 12 March 2026).

Adhesion to mammary epithelial cells is the first critical step in *S. aureus* intramammary infection (Figure 1).

Adhesion to host tissues is mediated by microbial surface components recognizing adhesive matrix molecules (MSCRAMMs), which enable *S. aureus* to attach to mammary epithelial cells and initiate colonization. Variability in the expression and regulation of these adhesins among strains may contribute to differences in invasion capacity, persistence, and disease outcome [27,28].

Once adhered, *S. aureus* can invade non-phagocytic mammary epithelial cells, a strategy that helps it evade host defenses and antimicrobial agents. Following adhesion, *S. aureus* can invade mammary epithelial cells, enabling evasion of host defenses and antimicrobial exposure. Notably, invasion capacity varies among strains and is influenced by regulatory systems such as *agr*, contributing to differences in persistence and clinical outcome. Experimental studies have demonstrated this variability, with certain strain types exhibiting higher invasion potential in vitro [29,30]. Some strains show variation in their adhesion/invasion rates depending on their *agr* type or regulatory background. Bonsaglia et al. (2025) observed that strains with *agr* group I (and certain MSCRAMM genotypes) had higher relative invasion rates in bMEC assays [28]. In bovine intramammary infection, the efficiency of adhesion and invasion may vary among strains, accounting in part for differences in disease severity, persistence, and response to treatment [7].

*Staphylococcus aureus* employs multiple virulence strategies, including adhesion, invasion, biofilm formation, toxin production, and immune evasion, which are summarized in Table 3.

A defining feature of *S. aureus* pathogenesis in bovine mastitis is its extensive arsenal of virulence factors that facilitate immune evasion, tissue damage, and persistence within the host [28]. Among these, Protein A (encoded by *spa*) plays a central role by binding to the Fc region of immunoglobulin G (IgG), thereby preventing effective opsonization and subsequent phagocytosis by neutrophils and macrophages [33]. This mechanism allows *S. aureus* to evade one of the host’s primary innate immune defenses and contributes to the establishment of persistent intramammary infections [29]. *S. aureus* produces a range of toxins that contribute to tissue damage and immune modulation, with leukocidins, particularly LukMF′ in ruminants, playing a key role in impairing host defense mechanisms through neutrophil lysis [7,30,32]. The coordinated action of these virulence factors enables *S. aureus* to both resist immune clearance and induce host tissue injury. Importantly, the expression of many of these factors is regulated by global regulatory systems such as *agr* and *sarA*, which modulate virulence gene expression in response to environmental signals [28,29]. This regulatory flexibility allows the pathogen to adapt to different stages of infection, transitioning from colonization to invasion and persistence. Overall, the interplay between immune evasion mechanisms and cytotoxic activity is central to the pathogenesis of *S. aureus* mastitis, contributing to its chronicity, recurrence, and poor response to antimicrobial therapy [7,9].

Biofilm formation (Figure 1) is a critical virulence strategy that significantly contributes to the persistence and treatment resistance of *S. aureus* in bovine mastitis [9]. Within the mammary gland, *S. aureus* can form structured, surface-associated microbial communities embedded in a self-produced extracellular polymeric matrix composed of polysaccharides, proteins, and extracellular DNA [35]. This biofilm mode of growth provides a protective niche that enhances bacterial survival under both host immune pressure and antimicrobial therapy [9,12].

The formation of biofilms in *S. aureus* is mediated by multiple genetic determinants, among which the icaADBC operon plays a central role by encoding enzymes responsible for the synthesis of polysaccharide intercellular adhesin (PIA), a key component of the biofilm matrix [9,35]. In addition to PIA-dependent mechanisms, biofilm formation can also occur through protein-mediated pathways involving surface-associated proteins such as biofilm-associated protein (Bap) and fibronectin-binding proteins (FnBPs), highlighting the redundancy and adaptability of this process [7,27]. This diversity in biofilm-forming mechanisms allows *S. aureus* strains to maintain persistence under varying environmental and host conditions.

Biofilm-associated bacteria exhibit markedly increased tolerance to antimicrobial agents compared to their planktonic counterparts [35]. This reduced susceptibility is attributed to multiple factors, including limited antibiotic penetration through the biofilm matrix, altered metabolic activity of bacteria within deeper biofilm layers, and the presence of persister cells that exhibit transient antibiotic tolerance [9]. Consequently, standard intramammary antimicrobial treatments often fail to achieve bacteriological cure in biofilm-associated infections, contributing to the chronic and recurrent nature of *S. aureus* mastitis [9].

In addition to conferring antimicrobial tolerance, biofilms play a crucial role in immune evasion by limiting immune-cell access and reducing the effectiveness of opsonization and phagocytosis [9,12,13]. The extracellular matrix can impede the access of immune cells such as neutrophils and macrophages, while also reducing the effectiveness of opsonization and phagocytosis [35]. Furthermore, biofilm-associated bacteria can modulate host immune responses, leading to a state of persistent low-grade inflammation that favors bacterial survival without triggering effective clearance [12,13].

Regulation of biofilm formation is tightly controlled by global regulatory systems, particularly the accessory gene regulator (*agr*) and staphylococcal accessory regulator (*sarA*) [7,35]. While *agr* is generally associated with the upregulation of toxin production and downregulation of surface adhesins, reduced agr activity has been linked to enhanced biofilm formation and persistence, a phenotype frequently observed in chronic infections [7,28]. This regulatory balance between planktonic virulence and biofilm-associated persistence highlights the adaptive capacity of *S. aureus* during different stages of infection [7,35]. Overall, biofilm formation represents a central mechanism underlying the chronicity, antimicrobial resistance, and therapeutic failure associated with *S. aureus* mastitis. Its multifactorial nature and tight regulatory control make it a key target for the development of novel therapeutic and preventive strategies.

Beyond extracellular defense mechanisms, *S. aureus* employs sophisticated strategies to evade host immunity and persist within mammary tissue, notably through intracellular survival and the formation of small-colony variants (SCVs) [34,35]. Following adhesion to mammary epithelial cells, *S. aureus* can invade and survive within non-phagocytic cells, including bovine mammary epithelial cells (bMECs), thereby escaping immune surveillance and reducing exposure to antimicrobial agents [6,29].

Intracellular persistence is primarily mediated by fibronectin-binding proteins (FnBPA and FnBPB), which facilitate bacterial uptake via interaction with host integrins, triggering endocytosis [28,35]. Once internalized, *S. aureus* can reside within intracellular compartments, where it adopts a low-metabolic state that enhances survival under hostile conditions, including oxidative stress and antibiotic exposure [28,29]. This intracellular niche acts as a reservoir for recurrent infection, allowing bacteria to evade both humoral and cellular immune responses.

A key adaptation associated with intracellular persistence is the emergence of SCVs, a slow-growing phenotype characterized by reduced metabolism, altered electron transport, and decreased expression of virulence factors [34,35]. SCVs exhibit increased tolerance to antibiotics, particularly those targeting actively dividing cells, and are less readily detected by conventional diagnostic methods [35]. In the context of bovine mastitis, SCVs have been implicated in chronic, subclinical infections that are difficult to eradicate and prone to relapse following treatment.

Importantly, SCVs demonstrate enhanced intracellular survival and can revert to the more virulent wild-type phenotype under favorable conditions, contributing to cyclical patterns of infection and clinical flare-ups [34,35]. This phenotypic switching highlights the dynamic adaptability of *S. aureus* within the host environment and complicates both diagnosis and treatment strategies [7,35]. In addition to intracellular survival and SCV formation, *S. aureus* employs multiple immune evasion mechanisms that interfere with host defense pathways. These include inhibition of complement activation, resistance to phagocytosis, and modulation of cytokine responses, which collectively impair effective immune clearance [7,33]. The combination of intracellular persistence, phenotypic variation, and immune modulation enables *S. aureus* to establish long-term infections within the mammary gland. The integration of intracellular survival, phenotypic switching, and immune modulation underpins the persistence of *S. aureus* within the mammary gland and its capacity to evade both host defenses and antimicrobial interventions [7,35]. Despite extensive characterization of individual virulence factors, a key knowledge gap remains in understanding how these mechanisms interact in vivo to determine infection outcomes, particularly the transition from acute to chronic or subclinical infection.

## 4. Clinical Manifestations

The clinical presentation of *S. aureus* bovine mastitis is notably heterogeneous, ranging from occasional acute episodes with visible udder inflammation and abnormal milk to the far more common chronic and subclinical forms that persist with few or no obvious external signs [36]. In practice, *S. aureus* is especially important because it frequently establishes long-lasting intramammary infections characterized by intermittent bacterial shedding, elevated somatic cell counts (SCC), and recurrent flare-ups, which complicate both diagnosis and herd-level control [36]. This subclinical persistence is of particular concern because infected cows may remain undetected for prolonged periods while continuing to serve as reservoirs of transmission within the herd. Beyond animal health, these infections also have important production consequences, as increased SCC and chronic inflammation are associated with reduced milk yield and deterioration of milk quality, including impaired technological properties relevant to dairy processing [37]. Together, these features make *S. aureus* mastitis a clinically deceptive but economically significant disease, in which apparently mild or silent infections can still exert substantial effects on udder health, milk quality, and control success.

*S. aureus* intramammary infections can manifest as either acute or chronic forms, although the latter predominates in dairy herds [36]. Acute mastitis caused by *S. aureus* is relatively uncommon but clinically significant, presenting with visible signs such as udder swelling, heat, pain, and abnormalities in milk, including clots, flakes, or discoloration. In severe cases, systemic signs such as fever, reduced appetite, and decreased milk production may also occur, reflecting a strong inflammatory response to bacterial toxins and rapid tissue damage. These acute episodes are often associated with the expression of virulence factors such as hemolysins and leukocidins, which contribute to epithelial and immune cell injury [7,36]. In contrast, chronic infections are far more typical of *S. aureus* mastitis and are characterized by persistent, often subclinical intramammary colonization. These infections may last for weeks, months, or even the entire lactation period, with intermittent bacterial shedding and periodic mild clinical flare-ups [36]. The chronicity of *S. aureus* infections is largely driven by persistence mechanisms such as intracellular survival and biofilm formation (see Section 3), resulting in poor therapeutic response and the establishment of long-term carrier animals that act as reservoirs of infection within the herd [37,38]. The distinction between acute and chronic forms is therefore not only clinical but also epidemiological and therapeutic, as chronic infections are more difficult to detect, less responsive to treatment, and play a central role in the persistence and spread of *S. aureus* within dairy populations [36,38].

Subclinical mastitis represents the most prevalent manifestation of *S. aureus* intramammary infection and constitutes a major challenge for effective herd management due to its largely unapparent nature. In these cases, infected cows typically exhibit no visible signs of udder inflammation or milk abnormalities, yet harbor persistent bacterial populations within the mammary gland. Despite the absence of overt clinical symptoms, subclinical infections are associated with sustained elevations in somatic cell count (SCC), reflecting ongoing inflammatory responses and tissue damage [36,37].

A defining feature of *S. aureus* subclinical mastitis is its ability to establish long-term persistence within the host. This persistence is facilitated by multiple mechanisms, including intracellular survival within mammary epithelial cells, evasion of immune recognition, and biofilm formation, all of which contribute to reduced bacterial clearance and limited antimicrobial efficacy [7,9,35]. Additionally, *S. aureus* infections are often characterized by intermittent bacterial shedding, meaning that infected quarters may test negative at certain time points despite ongoing infection. This phenomenon significantly complicates diagnosis and can lead to underestimation of herd prevalence [21,37].

Detection of subclinical *S. aureus* mastitis relies primarily on indirect and direct diagnostic approaches, each with inherent limitations. Somatic cell count is widely used as a screening tool; however, elevated SCC is not specific to *S. aureus* and may vary depending on factors such as stage of lactation, parity, and concurrent infections [36]. Bacteriological culture remains the reference method for pathogen identification, but its sensitivity is reduced by intermittent shedding and low bacterial loads. Molecular techniques, such as PCR-based assays, offer improved sensitivity and specificity, yet their implementation in routine herd monitoring may be constrained by cost and technical requirements [36]. Collectively, the silent nature of subclinical infections, combined with diagnostic limitations and the pathogen’s persistence strategies, makes *S. aureus* mastitis particularly difficult to detect and control, allowing infected animals to remain undiagnosed sources of transmission within dairy herds.

Infection of the mammary gland with *S. aureus* is consistently associated with elevated somatic cell count (SCC), which serves as a key indicator of intramammary inflammation and is widely used in both herd monitoring and milk quality assessment [37]. The increase in SCC primarily reflects the recruitment of neutrophils into the mammary gland in response to bacterial presence and toxin-mediated tissue injury. In subclinical *S. aureus* infections, SCC levels are often persistently elevated but may fluctuate over time, complicating interpretation at the individual cow level while still contributing to increased bulk tank SCC [38,39].

Beyond its diagnostic value, elevated SCC has significant implications for milk yield and composition. Chronic *S. aureus* infections are associated with reduced milk production due to damage to secretory epithelial cells and altered mammary gland function. In addition, inflammatory processes lead to changes in milk constituents, including decreased lactose and casein content and increased levels of serum proteins, enzymes, and ions such as sodium and chloride [40]. These compositional alterations negatively affect the nutritional value of milk as well as its technological properties, particularly in cheese production, where high SCC is linked to reduced coagulation efficiency and lower cheese yield [36,40,41,42].

Importantly, even in the absence of clinical signs, subclinical *S. aureus* infections can significantly impair milk quality at both the individual and herd levels. Persistently high SCC contributes to economic losses through decreased milk yield, penalties for poor milk quality, and reduced shelf life of dairy products [1,36,43]. Moreover, enzymes released from somatic cells, such as proteases and lipases, can degrade milk components during storage, further compromising product quality. Thus, the impact of *S. aureus* mastitis extends beyond animal health, representing a critical issue for dairy production efficiency and product quality [43].

## 5. Diagnosis

Accurate diagnosis of *S. aureus* mastitis is fundamental for effective treatment, control, and prevention strategies in dairy herds. However, detection of this pathogen remains particularly challenging due to its biological characteristics, including intermittent bacterial shedding, low bacterial loads in subclinical infections, and the ability to persist within host tissues. These factors can reduce the sensitivity of diagnostic methods and lead to an underestimation of infection prevalence [36,39,41].

A variety of diagnostic approaches are currently employed, ranging from conventional bacteriological culture to advanced molecular and rapid detection technologies. Each method presents distinct advantages and limitations in terms of sensitivity, specificity, cost, and applicability under field conditions. Consequently, accurate identification of *S. aureus* often requires a combined diagnostic approach that integrates multiple techniques to improve detection reliability and support informed herd management decisions [36,44].

Conventional bacteriological culture remains the reference standard for the diagnosis of *S. aureus* intramammary infections and is widely applied in both routine herd monitoring and clinical investigations [39]. Milk samples are typically aseptically collected and plated on general or selective media, such as blood agar, followed by identification based on colony morphology, hemolytic activity, and biochemical characteristics, including catalase and coagulase tests. This approach enables reliable species-level identification and allows subsequent antimicrobial susceptibility testing, which is essential for guiding therapeutic decisions [21,41,45].

Despite its widespread use, culture-based diagnosis has several important limitations, particularly in the context of *S. aureus* mastitis. The pathogen is often shed intermittently in milk, and bacterial loads may be low, especially in subclinical or chronic infections [37,39,41]. These factors can lead to false-negative results from single milk samples. In addition, prior antimicrobial treatment and improper sample handling may further reduce culture sensitivity. To mitigate these issues, repeated sampling or the use of composite samples from multiple milkings is often recommended to improve detection rates [36,41].

Another advantage of conventional culture is its role in epidemiological investigations, as isolates can be further characterized using phenotypic or genotypic methods to study transmission patterns within herds. However, the main drawbacks of this approach include the time required to obtain results, typically 24–72 h, and the need for laboratory infrastructure and trained personnel [39,46]. Nevertheless, due to its high specificity, cost-effectiveness, and ability to provide viable isolates, bacteriological culture remains a cornerstone of mastitis diagnosis and continues to be widely used in both research and field settings [46].

Molecular diagnostic methods have become increasingly important in the detection of *S. aureus* intramammary infections, offering improved sensitivity and faster turnaround times compared to conventional culture [47]. Polymerase chain reaction (PCR) assays are widely used to identify *S. aureus* by targeting species-specific genes such as nuc, spa, or femA, enabling rapid and accurate detection directly from milk samples. These methods are particularly valuable in cases where bacterial loads are low or when prior antimicrobial treatment has reduced culture viability [47].

Quantitative PCR (qPCR) further enhances diagnostic capability by allowing the quantification of bacterial DNA, providing an estimate of pathogen load that may be useful for assessing infection dynamics and severity [48]. In addition, multiplex PCR assays enable the simultaneous detection of multiple mastitis pathogens, improving diagnostic efficiency in herd-level screening programs. However, one important limitation of PCR-based methods is their inability to distinguish between DNA from viable and non-viable bacteria, which may lead to overestimation of active infections [48].

More advanced molecular approaches, such as sequencing technologies, are increasingly applied for detailed characterization of *S. aureus* isolates [49]. Whole-genome sequencing (WGS) allows high-resolution analysis of strain diversity, virulence factors, and antimicrobial resistance genes, supporting epidemiological investigations and outbreak tracing within and between dairy herds. Although these techniques provide valuable insights, their routine use in field diagnostics remains limited by cost, technical complexity, and the need for specialized bioinformatics expertise [36,49]. Overall, molecular diagnostics represent a powerful complement to conventional methods, particularly for improving detection sensitivity and enabling detailed characterization of *S. aureus*. However, their optimal use requires careful interpretation in conjunction with clinical findings and other diagnostic results.

Recent advances in diagnostic technologies have focused on the development of rapid, user-friendly tools for the on-farm detection of mastitis pathogens, including *S. aureus*. These approaches aim to overcome the limitations of conventional culture and laboratory-based molecular methods by providing faster results, enabling timely intervention, and improved herd management [50,51,52].

Biosensor-based systems represent a promising area of innovation in mastitis diagnostics. These devices integrate biological recognition elements, such as antibodies, nucleic acids, or aptamers, with signal transducers to detect specific bacterial components or host-derived biomarkers in milk. Various platforms, including electrochemical, optical, and immunosensors, have demonstrated the ability to detect *S. aureus* with high sensitivity and specificity, often within a short time frame. Some biosensors target bacterial DNA or toxins, while others focus on host inflammatory markers associated with infection [50].

In parallel, point-of-care (POC) diagnostic tools are being developed to allow rapid decision-making directly at the farm level [51]. These include lateral flow assays, microfluidic devices, and portable PCR systems, which can deliver results within minutes to a few hours without the need for extensive laboratory infrastructure. Such technologies have the potential to facilitate early detection of subclinical infections, guide selective treatment strategies, and reduce unnecessary antimicrobial use [51,52].

Despite their advantages, rapid diagnostic tools still face several challenges that limit widespread adoption [50]. These include variability in sensitivity under field conditions, limited validation across diverse herd settings, and cost considerations. Additionally, some assays may lack the ability to provide comprehensive information, such as antimicrobial susceptibility profiles [52,53,54]. Nevertheless, continued technological advancements and increasing demand for precision dairy farming are expected to drive the integration of biosensors and point-of-care diagnostics into routine mastitis management in the near future [50,52,53].

Recent advances in high-throughput sequencing have enabled the application of metagenomic approaches to bovine mastitis research, allowing the detection of *S. aureus* within complex milk microbial communities without prior culture [55,56]. While these methods provide valuable insights into microbiome composition and pathogen co-occurrence, reported frequency estimates remain highly variable due to differences in sequencing depth, sample processing, and bioinformatic pipelines. Moreover, the lack of standardized thresholds for clinical relevance limits the interpretation of these findings. Consequently, despite their clear potential, metagenomic approaches currently remain primarily research tools, with limited application in routine diagnostics and epidemiological surveillance. In contrast, conventional culture and PCR-based methods remain the mainstay of routine diagnostics, while more advanced approaches are largely restricted to research or specialized laboratory settings [55,56].

## 6. Treatment Challenges

The treatment of *S. aureus* mastitis remains one of the most challenging aspects of disease management in dairy cattle, largely due to the pathogen’s ability to evade host immune defenses and resist antimicrobial therapy [57]. Compared to other mastitis pathogens, *S. aureus* infections are associated with consistently lower bacteriological cure rates, particularly in chronic and subclinical cases, where infections often persist despite treatment. This reduced therapeutic success has important implications for animal health, antimicrobial stewardship, and dairy herd productivity [45].

Several biological and clinical factors contribute to the poor response of *S. aureus* mastitis to treatment. These include the capacity of the bacterium to form biofilms, persist intracellularly within mammary epithelial cells, and develop phenotypic variants such as small-colony variants, all of which reduce antimicrobial penetration and effectiveness [9,35]. In addition, infections are frequently well established by the time of detection, particularly in subclinical cases, further decreasing the likelihood of successful therapy [54].

The increasing prevalence of antimicrobial resistance among *S. aureus* isolates, including resistance to β-lactam antibiotics, further complicates treatment strategies and limits available therapeutic options. Consequently, treatment outcomes are often inconsistent, and infected cows may become chronic carriers, serving as reservoirs of infection within the herd. These challenges highlight the need for improved therapeutic approaches and reinforce the importance of prevention and early detection in controlling *S. aureus* mastitis [41].

Antimicrobial resistance in *S. aureus* has emerged as a major concern in the context of bovine mastitis, with increasing reports of both methicillin-resistant *S. aureus* (MRSA) and multidrug-resistant (MDR) strains in dairy herds worldwide. MRSA strains are characterized by the presence of the *mec*A or *mec*C genes, which encode an altered penicillin-binding protein (PBP2a) that confers resistance to β-lactam antibiotics, including penicillins and cephalosporins. Although historically associated with human healthcare settings, MRSA has now been identified in livestock, including dairy cattle, raising concerns about zoonotic transmission and public health implications [57,58]. Importantly, general reduced antimicrobial susceptibility, MDR, MRSA, and livestock-associated MRSA (LA-MRSA) represent distinct but related phenomena with different clinical, epidemiological, and zoonotic implications. Key antimicrobial resistance determinants reported in *S. aureus* from bovine mastitis are summarized in Table 4.

In addition to MRSA, MDR *S. aureus* strains, defined as isolates resistant to three or more antimicrobial classes, are increasingly reported in mastitis cases. Extensively drug-resistant (XDR) phenotypes have also been described, and although still rare, the potential emergence of pan-drug-resistant (PDR) *S. aureus*, defined as resistance to all available antimicrobial classes, represents an additional concern [62]. Resistance has been documented against commonly used antimicrobials such as β-lactams, tetracyclines, macrolides, and aminoglycosides. The emergence of these resistant phenotypes is largely driven by selective pressure from antimicrobial use in dairy production, as well as the ability of *S. aureus* to acquire and disseminate resistance genes through mobile genetic elements such as plasmids and transposons [7,59].

Recent studies indicate considerable geographic variation in resistance patterns, with higher prevalence of MRSA and MDR strains reported in regions with intensive dairy production and less regulated antimicrobial use. Although the overall prevalence of MRSA in bovine mastitis remains relatively low compared to human infections, its detection in milk and dairy environments is of growing concern due to the potential for transmission along the food chain and between animals and humans. Furthermore, MDR strains complicate therapeutic decision-making and are associated with reduced treatment efficacy and increased likelihood of persistent infections [60]. Notably, the reported prevalence of MRSA in bovine mastitis remains generally lower than in human clinical settings, but regional variability is substantial, with higher rates observed in intensive production systems and areas with less regulated antimicrobial use. Overall, the rise of antimicrobial resistance in *S. aureus* highlights the need for prudent antimicrobial use, continuous surveillance, and the development of alternative control strategies to mitigate the spread of resistant strains in dairy systems.

Conventional intramammary antimicrobial therapy remains a primary approach for the treatment of bovine mastitis; however, its efficacy against *S. aureus* infections is often limited. Bacteriological cure rates for *S. aureus* mastitis are generally lower than those observed for other major mastitis pathogens, particularly in chronic and subclinical cases. Reported cure rates vary widely but are frequently below 50%, especially in lactating cows, reflecting the difficulty of eliminating established infections [45,63]. Importantly, reported cure rates vary considerably across studies, reflecting differences in antimicrobial protocols, stage of infection, and strain-specific factors. This variability emphasizes the lack of standardized treatment strategies and highlights the need for more evidence-based, targeted therapeutic approaches.

Reduced effectiveness of intramammary therapy is associated with several well-defined factors, including limited antimicrobial penetration into fibrotic tissue and abscesses within the mammary gland, delayed initiation of treatment after infections become established, and variability in antimicrobial susceptibility among *S. aureus* isolates [41,53]. In addition, biofilm formation and intracellular persistence further reduce antimicrobial efficacy, contributing to treatment failure and chronic infection [41,60].

A major factor underlying the persistence and recurrence of *S. aureus* mastitis is the pathogen’s ability to evade both host immune responses and antimicrobial treatment through biofilm formation and intracellular survival [7,9,35]. Biofilm formation (see Section 3) further reduces antimicrobial penetration and contributes to treatment failure in established infections [54,64].

In addition, intracellular persistence further limits antimicrobial efficacy (see Section 3), contributing to treatment failure and relapse. Furthermore, the formation of SCVs, which are slow-growing and less metabolically active, is associated with enhanced intracellular survival and increased resistance to antimicrobial therapy [34,64].

## 7. Prevention and Control

**Figure 2:**
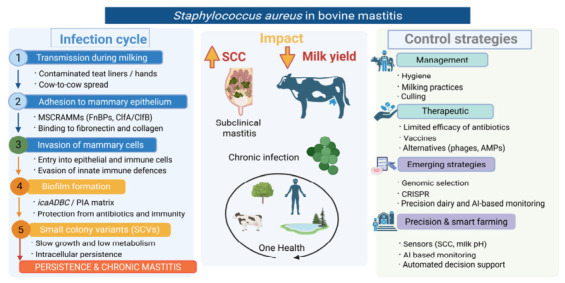
Infection cycle and control strategies of *Staphylococcus aureus* in bovine mastitis. The infection cycle includes transmission during milking, adhesion to the mammary epithelium, invasion of host cells, biofilm formation, and the emergence of small-colony variants (SCVs), leading to persistent infection. These processes result in increased somatic cell count (SCC) and reduced milk yield, contributing to subclinical and chronic mastitis. Control strategies include management practices (hygiene, milking procedures, culling), therapeutic interventions with limited antibiotic efficacy, and emerging approaches such as vaccines, bacteriophages, antimicrobial peptides (AMPs), genomic selection, CRISPR-based tools, and precision dairy technologies, including AI-based monitoring. Abbreviations: MSCRAMMs, microbial surface components recognizing adhesive matrix molecules; FnBPs, fibronectin-binding proteins; ClfA/ClfB, clumping factors A/B; icaABCD, intercellular adhesion gene cluster; PIA, polysaccharide intercellular adhesin; SCVs, small colony variants; SCC, somatic cell count; AMPs, antimicrobial peptides; CRISPR, clustered regularly interspaced short palindromic repeats. Created in BioRender. Nadas, N. (2026) https://BioRender.com/dzl968q (accessed on 12 March 2026).

Effective control of *S. aureus* mastitis requires an integrated understanding of its infection cycle, clinical impact, and available intervention strategies [65]. As summarized in Figure 2, disease persistence results from a combination of transmission dynamics, host–pathogen interactions, and limitations of current therapeutic approaches, highlighting the need for coordinated management, therapeutic, and emerging strategies.

Effective prevention and control of *S. aureus* mastitis rely heavily on strict milking hygiene and sound herd management practices, as the pathogen is primarily transmitted during milking. Contagious spread occurs mainly via contaminated milking equipment, milkers’ hands, and udder cloths, making hygiene at this stage critical for limiting within-herd transmission [41,53].

Key preventive measures include proper pre- and post-milking teat disinfection, the use of individual towels for udder preparation, and the consistent wearing and sanitation of gloves by milkers. Post-milking teat dipping is particularly effective in reducing new intramammary infections by eliminating bacteria present on the teat skin immediately after milking. In addition, maintaining well-functioning milking equipment and ensuring appropriate milking machine settings are essential to prevent teat-end damage, which can predispose cows to infection [41,66].

Segregation or separate milking of infected cows is another important strategy to reduce transmission, especially in herds with a high prevalence of *S. aureus*. Chronically infected animals may serve as persistent reservoirs and, in some cases, culling may be necessary when treatment is unsuccessful. Routine monitoring through SCC and bacteriological testing supports early identification of infected animals and helps guide management decisions [63]. Consistent implementation of milking hygiene protocols and management practices is essential for controlling the spread of *S. aureus* within dairy herds, as prevention remains more effective than treatment for this pathogen.

Selective culling of chronically infected cows is a key component of control programs for *S. aureus* mastitis, particularly in herds where infection persists despite appropriate treatment and hygiene measures. Due to the pathogen’s ability to establish long-term intramammary infections with low cure rates, chronically infected animals often act as continuous reservoirs of transmission, contributing to the spread of infection within the herd during milking [41,63].

Culling decisions are typically based on a combination of factors, including persistently elevated SCC, repeated positive bacteriological cultures for *S. aureus*, poor response to antimicrobial therapy, and reduced milk production. Cows with multiple infected quarters or long-standing infections are particularly unlikely to achieve bacteriological cure and are therefore strong candidates for removal from the herd. In such cases, selective culling can significantly reduce infection pressure and improve overall udder health at the herd level [66].

Economic considerations also play an important role in culling decisions. While removing infected animals incurs immediate costs, these may be offset by long-term benefits, including reduced transmission, improved milk quality, and lower treatment expenses. In well-managed herds, targeted culling of *S. aureus*-infected cows is an effective strategy for controlling contagious mastitis and reducing bulk tank SCC [41,63]. Selective culling should be viewed as part of an integrated mastitis control program, particularly for managing chronic *S. aureus* infections that are unlikely to respond to conventional therapy.

Vaccination against *S. aureus* mastitis has long been pursued as a preventive strategy, but overall results have been mixed, and no vaccine has delivered consistently strong, sterilizing protection under field conditions. Recent reviews conclude that mastitis vaccines can sometimes reduce clinical severity, somatic cell count, or the duration of infection, yet their ability to reliably prevent new *S. aureus* intramammary infections or eliminate established infections remains limited [65].

Some successes have nevertheless been reported. Experimental and field studies have shown partial benefits from whole-cell bacterins or multicomponent vaccines, including reduced severity of mastitis, lower bacterial shedding, and in some cases lower incidence of staphylococcal mastitis. An older challenge study by Leitner et al. reported substantial protection in vaccinated cows, while a recent field study by Vidlund et al. found that one surface-protein vaccine candidate significantly reduced the incidence of staphylococcal mastitis under natural exposure, suggesting that improvement is possible with better antigen selection [67,68].

However, most vaccination attempts have fallen short of expectations. Systematic review and meta-analytic evidence indicates that protection is often incomplete, inconsistent across studies, and highly dependent on herd conditions, vaccine composition, adjuvant choice, and study design. Commercial vaccines such as Startvac have shown variable performance, with some trials reporting modest benefits and others finding limited effect on new infection rates or bacteriological cure [69,70].

Several factors help explain these failures. *S. aureus* is antigenically diverse, can persist intracellularly, forms biofilms, and often causes chronic rather than acute infections, all of which reduce the effectiveness of antibody-dominated vaccine responses [69]. In addition, the mammary gland has distinctive immunobiological constraints, and natural infection itself does not generate strong sterilizing immunity, which suggests that successful vaccination will need to induce immune mechanisms that are qualitatively better than those produced by infection alone [65]. The literature supports a cautious conclusion: vaccination may serve as an adjunct to herd control programs by reducing disease expression or transmission pressure, but it cannot yet replace core measures such as milking hygiene, segregation, monitoring, and selective culling. At present, the main lesson from both successes and failures is that future vaccines will likely need better-defined protective antigens, stronger mucosal or cell-mediated immune responses, and more realistic field validation before they can provide dependable control of *S. aureus* mastitis.

Genetic selection for mastitis resistance has gained increasing attention as a sustainable strategy to reduce the impact of *S. aureus* infections in dairy herds. Unlike therapeutic or management interventions, breeding for resistance offers a long-term solution by enhancing the cow’s inherent ability to prevent or control intramammary infections. Recent genomic approaches have significantly improved the feasibility of selecting for disease resistance traits, particularly through the use of SCC and genomic breeding values as indirect indicators of mastitis resistance [71].

Recent advances in genomics have improved the identification of genetic markers associated with mastitis resistance [72]. Genome-wide association studies (GWAS) and genomic selection approaches have identified candidate genes involved in immune response pathways, including TLR2, TLR4, and CXCR1, which are associated with pathogen recognition and inflammatory responses [71,72]. These tools enable more accurate estimation of breeding values and allow earlier selection of animals with improved resistance to mastitis, including infections caused by *S. aureus* [72].

Despite these advances, breeding for mastitis resistance remains challenging due to the complex and polygenic nature of the trait. Heritability is generally low to moderate, and resistance is influenced by environmental and management factors. Furthermore, selection based solely on SCC may not fully capture resistance to specific pathogens such as *S. aureus*, and there is a need to balance health traits with production traits to avoid an unfavorable trade-off [71]. Genetic selection represents a promising long-term strategy for mastitis control. When integrated with improved management practices and disease monitoring, breeding for resistance traits can contribute to reduced disease prevalence, improved animal welfare, and decreased reliance on antimicrobial treatments [22,72].

## 8. Alternative and Emerging Approaches

The persistent and often refractory nature of *S. aureus* bovine mastitis, driven by antimicrobial resistance, biofilm formation, and intracellular survival, has highlighted the limitations of conventional therapeutic strategies. As a result, there is growing interest in alternative and emerging approaches that aim to improve treatment efficacy, reduce antibiotic use, and support more sustainable mastitis control. These strategies include biological therapies, immunomodulation, and novel antimicrobial technologies, many of which are still under investigation but show considerable promise for future application in dairy practice [22,64].

Bacteriophage (phage) therapy has emerged as a promising alternative strategy for the control of *S. aureus* bovine mastitis, particularly in the context of increasing antimicrobial resistance and the limited efficacy of conventional therapies [15]. Phages are viruses that specifically infect and lyse bacterial cells, offering a highly targeted approach that can reduce pathogen load without disrupting the normal microbiota. Several in vitro and in vivo studies have demonstrated the ability of lytic phages to effectively reduce *S. aureus* populations, including strains embedded within biofilms, which are typically resistant to antibiotic treatment. Moreover, phages can replicate at the site of infection, potentially enhancing their therapeutic efficacy over time [15,73].

Despite these advantages, the application of phage therapy in dairy cattle faces several challenges. These include the narrow host range of individual phages, the potential for bacterial resistance to phages, regulatory hurdles, and the need for well-designed delivery systems that can maintain phage stability within the mammary gland environment [15,74]. Additionally, variability in treatment outcomes under field conditions highlights the importance of further research to optimize phage selection, formulation, and dosing strategies. Nevertheless, advances in phage engineering, cocktail formulations, and combined therapies (e.g., phages with antibiotics or enzymes) are expected to enhance their practical applicability, positioning bacteriophage therapy as a promising component of future mastitis control programs [74].

Antimicrobial peptides (AMPs) and plant-derived compounds are increasingly being investigated as alternative or complementary strategies for the control of *S. aureus* bovine mastitis. These approaches are particularly attractive due to their broad-spectrum activity, diverse mechanisms of action, and lower propensity for inducing resistance compared to conventional antibiotics. AMPs are small, naturally occurring molecules that form part of the innate immune defense in many organisms [75,76,77]. They exert their antimicrobial effects primarily through disruption of bacterial cell membranes, leading to rapid cell death. In the context of *S. aureus* mastitis, several AMPs, including defensins, cathelicidins, and synthetic analogs, have demonstrated strong activity against both planktonic cells and biofilm-associated bacteria. Additionally, some AMPs exhibit immunomodulatory properties, enhancing host defense mechanisms and promoting the resolution of infection [75,76].

Plant-derived compounds, including essential oils, flavonoids, tannins, and alkaloids, have also shown significant anti-staphylococcal activity [77]. Other natural products, such as propolis, have also demonstrated antimicrobial and anti-biofilm activity against *S. aureus* in preliminary studies, although further validation in vivo is required [78]. These compounds can interfere with bacterial cell wall integrity, inhibit enzyme systems, disrupt quorum sensing, and impair biofilm formation. Extracts from plants such as *Thymus vulgaris*, *Origanum vulgare*, and *Melaleuca alternifolia* have been reported to inhibit *S. aureus* growth and reduce virulence factor expression. Importantly, some phytochemicals have demonstrated synergistic effects when used in combination with antibiotics, potentially restoring the efficacy of drugs against resistant strains [78,79].

Despite their promise, several limitations remain. These include variability in activity depending on compound composition, potential cytotoxicity at higher concentrations, stability issues, and challenges related to formulation and delivery within the mammary gland [77]. Moreover, ongoing research into peptide engineering, nanoformulations, and standardized plant extracts is expected to enhance their therapeutic potential. As such, AMPs and plant-derived compounds represent a promising and versatile component of future mastitis management strategies [77,78,80].

Nanotechnology-based delivery systems are gaining increasing attention as innovative tools to enhance the treatment of *S. aureus* bovine mastitis [81]. These systems are designed to improve the pharmacokinetic and pharmacodynamic properties of antimicrobial agents, enabling more effective delivery to the site of infection while minimizing systemic exposure and drug residues in milk. By encapsulating antibiotics or alternative therapeutics within nanoparticles, it is possible to enhance drug stability, control release profiles, and increase penetration into infected mammary tissue [81,82].

A key advantage of nanocarriers is their ability to overcome major barriers to *S. aureus* infections, particularly biofilm formation and intracellular persistence [82]. Nanoparticles, such as liposomes, polymeric nanoparticles, solid lipid nanoparticles, and nanoemulsions, can facilitate deeper penetration into biofilms and improve intracellular delivery of antimicrobial agents, thereby increasing their efficacy against persistent bacterial populations. In some cases, nanoparticles themselves (e.g., silver or zinc oxide nanoparticles) also exhibit intrinsic antimicrobial activity through mechanisms such as oxidative stress induction and membrane disruption [81,83]. Additionally, nanotechnology enables the co-delivery of multiple agents, such as antibiotics combined with anti-biofilm compounds, antimicrobial peptides, or quorum-sensing inhibitors, thereby producing synergistic effects. Targeted delivery strategies, including ligand-functionalized nanoparticles, are also being explored to enhance specificity for infected tissues and reduce off-target effects [81,82].

Despite their considerable potential, the practical implementation of nanotechnology-based systems in dairy medicine is still constrained by several factors. Key issues include uncertainties related to safety, biocompatibility, environmental implications, regulatory frameworks, and economic feasibility [82]. In addition, evidence from large-scale in vivo studies in dairy cattle remains limited. Nonetheless, ongoing progress in nanomaterial engineering, together with deeper insights into host–pathogen interactions, is likely to facilitate the future adoption of these technologies in mastitis management [81].

CRISPR-Cas–based technologies represent a novel and highly specific approach for the control of S. aureus in bovine mastitis [84]. Originally identified as an adaptive immune system in bacteria, CRISPR-Cas systems have been adapted as powerful tools for targeted genome editing and antimicrobial applications. In this context, CRISPR-based antimicrobials can be engineered to selectively target and cleave essential bacterial genes or antimicrobial resistance determinants, leading to precise elimination of pathogenic strains while sparing beneficial microbiota [84].

One of the most promising applications involves the use of CRISPR-Cas systems delivered via bacteriophages or plasmid vectors to specifically target *S. aureus* within the mammary gland [85]. This strategy allows for sequence-specific killing, including the removal of resistance genes such as *mec*A, which is responsible for methicillin resistance. Additionally, CRISPR interference (CRISPRi) approaches can be used to silence virulence factors, thereby reducing bacterial pathogenicity without necessarily killing the organism, which may help limit selective pressure for resistance [85,86]. Beyond direct antimicrobial applications, genomic interventions may also contribute to long-term control strategies. Advances in host genomics and gene editing raise the possibility of breeding or engineering dairy cattle with enhanced resistance to mastitis through selection or modification of genes involved in immune responses, udder integrity, and pathogen recognition [87].

However, the application of CRISPR-based technologies in veterinary medicine remains at an early stage, with challenges including efficient and safe delivery systems, potential off-target effects, regulatory and ethical considerations, and public acceptance [85]. Despite promising experimental outcomes, most emerging approaches face significant barriers to practical implementation, including limited validation under field conditions. Consequently, their immediate applicability in routine dairy practice remains restricted. Among these approaches, bacteriophage therapy and antimicrobial peptides have demonstrated efficacy in in vivo animal models, whereas phytogenic compounds have shown mainly in vitro and limited in vivo activity, nanotechnology-based systems remain largely experimental, whereas CRISPR-based strategies are still in early developmental stages [15,73,81,86].

## 9. Zoonotic and Public Health Relevance

*S. aureus* is not only a leading etiological agent of bovine mastitis but also an important zoonotic pathogen with significant public health implications [88]. Its capacity to circulate between animals, humans, and the environment, together with the increasing emergence of antimicrobial-resistant strains, increases the need to address mastitis within a broader One Health perspective rather than as an isolated veterinary issue [88].

Moreover, *S. aureus* is a well-established foodborne pathogen associated with the consumption of contaminated milk and dairy products, particularly those derived from raw or improperly handled milk [89]. In cases of bovine mastitis, infected cows can shed the bacterium directly into milk, leading to contamination at the primary production level. When hygienic milking practices, storage conditions, or pasteurization processes are inadequate, *S. aureus* may proliferate and produce heat-stable enterotoxins that can remain biologically active even after thermal processing, thereby posing a persistent risk to consumer health [89,90].

These enterotoxins are responsible for staphylococcal food poisoning, a condition characterized by the rapid onset of symptoms such as nausea, vomiting, abdominal cramps, and sometimes diarrhea. Importantly, because the toxins are heat- and proteolytic-enzyme resistant, the risk persists even when viable bacteria are no longer present in the final product. Dairy items such as raw milk, soft cheeses, and other minimally processed products are particularly susceptible to contamination and toxin accumulation [90,91].

The public health significance of *S. aureus* in dairy production is further amplified by the potential presence of antimicrobial-resistant strains, including MRSA, which may be transmitted through the food chain or via direct contact with animals. Consequently, strict adherence to hygiene standards, effective mastitis control programs, and proper milk processing are essential to reduce the risk of foodborne transmission and protect consumer health [88,89,90]. MRSA of bovine origin represents an important interface between animal and human health, particularly in occupational settings. Dairy cattle can act as reservoirs of MRSA, with transmission occurring during milking, animal handling, or contact with contaminated equipment and farm environments. Individuals in close and repeated contact with infected or colonized animals, such as farmers, veterinarians, and farm workers, are therefore at increased risk of colonization and, in some cases, clinical infection [92].

Livestock-associated MRSA (LA-MRSA), including lineages such as clonal complex 398 (CC398), has been widely reported in dairy herds and is of particular concern due to its ability to colonize humans asymptomatically while retaining pathogenic potential [93]. Although colonization is often transient, it can serve as a source of further transmission within the community or healthcare settings, especially if biosecurity and hygiene measures are inadequate. In some cases, MRSA strains originating from livestock have been associated with skin and soft tissue infections, and more rarely, invasive disease in humans [93,94].

The occupational risk is influenced by factors such as herd infection status, intensity of animal contact, use of personal protective measures, and adherence to hygiene protocols [95]. Moreover, the presence of antimicrobial resistance genes complicates treatment options and raises concerns about the dissemination of resistant strains across the animal–human interface. These considerations highlight the importance of surveillance, infection control practices, and a One Health approach to mitigate the spread of MRSA between cattle and humans [94,95].

The epidemiology and control of *S. aureus* mastitis are best understood within a One Health framework, which recognizes the interconnectedness of animal health, human health, and the environment. *S. aureus*, including antimicrobial-resistant strains such as MRSA, can circulate between dairy cattle, humans, and farm environments, creating multiple pathways for transmission and persistence [96]. This interconnected cycle highlights the limitations of addressing mastitis solely at the herd level without considering broader public health implications [96,97].

From a One Health perspective, mastitis control strategies must integrate prudent antimicrobial use, effective infection prevention measures, and continuous surveillance across both veterinary and human sectors [97]. Reducing antibiotic usage in dairy production is particularly important to limit the selection and spread of resistant strains that may impact human medicine. At the same time, improving farm hygiene, milking practices, and biosecurity can decrease pathogen transmission within herds and reduce occupational exposure [97,98].

Environmental factors, including manure management, water contamination, and farm waste, also play a role in the dissemination of *S. aureus* and resistance genes [99]. Therefore, coordinated efforts involving veterinarians, farmers, public health professionals, and policymakers are essential to develop sustainable control strategies. By adopting a One Health approach, it becomes possible to more effectively mitigate the impact of *S. aureus* mastitis while safeguarding both animal productivity and human health [99].

## 10. Future Directions

Despite significant advances in understanding the pathogenesis, epidemiology, and control of *S. aureus* bovine mastitis, the disease continues to pose substantial challenges to dairy production and public health. The persistence of infections, the emergence of antimicrobial resistance, and the limitations of current diagnostic and therapeutic approaches highlight the need for continued innovation [7,11]. Future research is expected to focus on the development of more effective, targeted, and sustainable strategies, including advanced diagnostics, alternative therapeutics, precision livestock farming tools, and integrated herd management systems [86]. Interdisciplinary approaches within a One Health framework will be essential to address the complex interactions between host, pathogen, and environment and to improve long-term mastitis control [96,97]. A major priority for future research is the integration of mechanistic insights with applied field studies to bridge the gap between experimental findings and practical mastitis control strategies [36,86].

The complex and multifactorial nature of *S. aureus* bovine mastitis necessitates integrated control strategies that combine multiple complementary approaches rather than relying on single interventions. Effective control requires the coordination of improved diagnostics, targeted treatment protocols, strict milking hygiene, biosecurity measures, and herd-level management practices. In particular, early detection of subclinical infections, combined with selective therapy and culling policies, can significantly reduce transmission within herds [29,36].

In addition, integrated strategies should incorporate advances in alternative therapeutics, including bacteriophages, antimicrobial peptides, and nanotechnology-based systems, alongside prudent antimicrobial use to limit the development of resistance. Genetic selection for mastitis-resistant animals and the application of precision dairy farming technologies for real-time monitoring of udder health may further enhance control efforts [61]. Successful implementation of these approaches depends on farmer education, veterinary guidance, and continuous surveillance systems that enable timely intervention and evaluation of control measures, ultimately improving disease control and dairy production sustainability [100,101].

These strategies should include selective antimicrobial use guided by susceptibility testing, alongside non-antibiotic alternatives where appropriate. Long-term measures, such as culling chronically infected animals and improving housing, milking systems, and monitoring programs, are essential to reduce infection pressure within herds. Advances in precision dairy technologies and genetic selection for resistance traits may further support these efforts [41]. Sustained collaboration between farmers, veterinarians, and researchers, together with continuous evaluation of management practices, is critical to achieve durable reductions in *S. aureus* prevalence and to promote responsible antimicrobial stewardship [36]. Despite extensive research, important gaps remain in understanding the interactions between *S. aureus* and the host immune system in bovine mastitis. In particular, the mechanisms underlying the transition from acute infection to chronic and subclinical persistence are not fully elucidated [36,86]. Key questions include how *S. aureus* adapts to the intramammary environment, evades immune responses, and establishes long-term survival through intracellular persistence, biofilm formation, and the development of small colony variants (SCVs) [102]. Additionally, variability in host susceptibility suggests that genetic, immunological, and physiological factors play a significant role in disease outcome, yet these determinants are not completely understood [71,72]. The specific immune pathways that confer protection versus those associated with ineffective or dysregulated responses require further clarification. A deeper understanding of these mechanisms is essential for the development of more effective vaccines, immunotherapies, and targeted interventions [103].

Advances in omics technologies, including genomics, transcriptomics, proteomics, and metabolomics, provide new opportunities to investigatehost-pathogen dynamics at a systems level [104]. However, integrating these data into practical applications for mastitis control remains a challenge. Addressing these research gaps will be critical for designing innovative strategies that can disrupt persistent infections and improve long-term disease control [104].

The increasing prevalence of antimicrobial resistance in *S. aureus*, including multidrug-resistant and methicillin-resistant strains, highlights the critical importance of antibiotic stewardship in bovine mastitis management [11,61]. The widespread and, in some cases, indiscriminate use of antibiotics in dairy production has contributed to the selection of resistant pathogens, reducing treatment efficacy and posing risks to both animal and public health. Consequently, there is a growing need to optimize antimicrobial use through evidence-based protocols, including selective therapy, appropriate drug choice and dosing, and reliance on culture and susceptibility testing [29].

In parallel, the development and implementation of sustainable alternatives to antibiotics are becoming essential components of future control strategies. Approaches such as bacteriophage therapy, antimicrobial peptides, plant-derived compounds, and nanotechnology-based delivery systems offer promising avenues to reduce dependence on conventional antimicrobials while maintaining therapeutic effectiveness [15,76,81,86]. Preventive measures, including improved herd management, vaccination strategies, and genetic selection for disease resistance, also play a key role in minimizing the need for antimicrobial interventions [104]. Together, responsible antibiotic use and the integration of alternative strategies are fundamental to preserving antimicrobial efficacy, limiting the spread of resistance, and ensuring the long-term sustainability of dairy production systems within a One Health context [98,104].

## 11. Conclusions

*Staphylococcus aureus* remains one of the most important and challenging pathogens in bovine mastitis due to its remarkable ability to establish persistent intramammary infections, evade host immune defenses, and withstand conventional antimicrobial therapies. Its impact extends beyond clinical disease, as subclinical infections play a major role in reduced milk yield, impaired milk quality, and sustained transmission within dairy herds, representing both an economic and animal health burden.

Effective control of *S. aureus* mastitis requires a multifaceted and integrated approach. Core measures such as strict milking hygiene, biosecurity protocols, and selective culling remain fundamental for limiting transmission. At the same time, advances in genetic selection for mastitis resistance offer promising long-term strategies to enhance herd resilience. However, the limited efficacy of conventional antibiotic therapy, particularly in the context of biofilm formation, intracellular persistence, and antimicrobial resistance, highlights the urgent need for alternative and complementary approaches, including vaccines, bacteriophage therapy, antimicrobial peptides, and nanotechnology-based interventions.

Looking forward, sustainable management of *S. aureus* mastitis will depend on the coordinated integration of preventive, therapeutic, and genetic strategies, supported by continued research into host–pathogen interactions and the development of effective, evidence-based alternatives to conventional treatments. Importantly, control efforts should be framed within a One Health perspective, acknowledging the interconnected risks to animal, human, and environmental health, particularly in relation to antimicrobial resistance and zoonotic transmission. In parallel, the adoption of precision dairy technologies, including sensor-based monitoring systems and artificial intelligence-driven decision support tools, offers significant potential to enable early detection, optimize treatment decisions, and improve herd-level disease management.

## Figures and Tables

**Table 1:** Comparative overview of recent reviews on *Staphylococcus aureus* in bovine mastitis and the distinct contribution of the present study.

Reference	Year	Scope	Key Limitations	Distinct Contribution of This Review
[7]	2022	Pathogenesis and strain diversity	Does not integrate virulence mechanisms with treatment outcomes or control strategies	Integrates virulence mechanisms with therapeutic failure and herd-level control
[11]	2024	Antimicrobial resistance and management	Primarily focused on resistance, with limited discussion of persistence mechanisms	Links resistance with biofilm formation, intracellular survival, and chronic infection
[6]	2024	General mastitis overview	Broad scope with limited pathogen-specific depth	Provides *S. aureus*-specific synthesis with detailed mechanistic and clinical integration
[15]	2023	Bacteriophage therapy	Focus restricted to a single alternative approach	Critically compares multiple emerging therapies (phage, AMPs, nanotechnology, CRISPR)
[16]	2020	Risk factors and treatment strategies	Does not incorporate recent molecular and genomic insights	Incorporates recent advances in genomics, diagnostics, and resistance
Present review	2026	Integrated analysis	—	Provides a unified framework linking persistence mechanisms, antimicrobial resistance, and translational limitations, with emphasis on clinical relevance and future research gaps

**Table 2:** Geographic distribution, prevalence, and strain characteristics of *Staphylococcus aureus* associated with bovine mastitis based on representative studies.

Country/Region	Prevalence (%)	Sample Type	Strain/Genotype (If Reported)	Key Notes	Reference
Ethiopia	10–66.6	Milk (clinical/subclinical mastitis)	Not specified	High variability across regions	[17]
Pakistan	43	Milk (clinical and subclinical cases)	Not specified	High prevalence in mastitic cows	[18]
Europe (multi-country)	1–10 (low SCC herds); up to 50–75 in poorly managed herds	Milk samples	CC97, CC8 (reported in some studies)	Strong association with herd hygiene	[6,8]
Global meta-analysis	25–28	Multiple studies	Various	Overall global prevalence estimate	[19]

**Table 3:** Summary of key pathogenic mechanisms and virulence factors of *Staphylococcus aureus* in bovine mastitis.

Mechanism/Process	Key Molecules/Factors	Functional Role in Mastitis	Representative References
Adhesion to host tissue	FnBPA, FnBPB, ClfA/ClfB, Cna, Eno	Mediates attachment to fibronectin, fibrinogen, and collagen, initiating colonization of mammary epithelium	[27,28]
Invasion/Internalization	FnBP–fibronectin–integrin interaction	Enables bacterial entry into mammary epithelial cells, protecting from immune defenses	[28]
Biofilm formation/persistence	icaA, icaD, Bap, PIA	Produces extracellular matrix protecting bacteria from antimicrobials and immune attack	[31]
Toxins/Cytolysins	α-hemolysin (hla), β-hemolysin (hlb), γ-hemolysin (hlg), LukMF′	Causes cell lysis, inflammation, tissue damage	[32,31]
Immune evasion	Protein A (spa), capsule (cap5/8), staphylococcal complement inhibitor (SCIN)	Prevent opsonization and complement activation; hinder phagocytosis	[33]
Intracellular persistence SCVs	Small-colony variants, altered metabolism	Allow chronic infection and antibiotic tolerance	[34]

Abbreviations: FnBPA/FnBPB, fibronectin-binding proteins A/B; ClfA/ClfB, clumping factors A/B; Cna, collagen adhesin; Eno, enolase; FnBP, fibronectin-binding protein; PIA, polysaccharide intercellular adhesin; icaA/icaD, intercellular adhesion genes; Bap, biofilm-associated protein; hla/hlb/hlg, hemolysin genes; LukMF′, leukocidin; SCIN, staphylococcal complement inhibitor; SCVs, small colony variants.

**Table 4:** Antimicrobial resistance genes and associated mobile genetic elements identified in *Staphylococcus aureus* from bovine mastitis.

Gene (ARG)	Resistance Phenotype	Mobile Element	Reported Strains/Notes	References
*mecA*, *mecC*	Methicillin resistance (MRSA)	SCCmec (staphylococcal cassette chromosome)	Detected in livestock-associated MRSA (e.g., CC97, CC398)	[20,47,57,58,59,60]
*blaZ*	β-lactam resistance	Plasmids (occasionally transposons)	Common in penicillin-resistant isolates from bovine mastitis	[10,11,14,61]
*tetK*	Tetracycline resistance	Plasmids	Frequently reported in bovine isolates	[10,14,61]
*tetM*	Tetracycline resistance	Transposons (e.g., Tn916-like elements)	Associated with transferable resistance	[10,14,61]
*ermA*, *ermC*	Macrolide–lincosamide–streptogramin B (MLSB) resistance	Plasmids and transposons	Linked to inducible or constitutive MLSB resistance phenotype	[10,14,61]

Abbreviations: ARG, antimicrobial resistance gene.

## Data Availability

No new data were created or analyzed in this study. Data sharing is not applicable to this article.
